# mSphere of Influence: an Inupiat Journey in Science

**DOI:** 10.1128/msphere.00595-19

**Published:** 2019-09-18

**Authors:** Kathryn C. A. Milligan-McClellan

**Affiliations:** a University of Alaska Anchorage, Anchorage, Alaska, USA

**Keywords:** Inupiaq, host-microbe, host-pathogen interactions, microbiota

## Abstract

Dr. Kathryn Milligan-McClellan works in the field of host-microbe interactions. In this mSphere of Influence article, she reflects on the people and scientific ideas that influenced her journey from a small town in Alaska to a faculty position at the University of Alaska Anchorage.

## COMMENTARY

I look down at my blueberry stained fingers holding my bag of carefully collected tundra tea and then quickly survey the landscape to keep an eye on my dad, who is scouring the valleys for aqpiks on this land that has provided for our ancestors for hundreds of generations, and my kids, who are patiently picking berries while trying not to get caught eating them. In this moment, I am not Dr. Kat, assistant professor, microbiology researcher, mentor; I am Kathy, daughter, mom, Inupiat, gatherer.

My career has been a precarious balance of the two versions of my being. Raised in Kotzebue, Alaska, I was taught to respect the land but also taught that being called Native is an insult. I was taught Inupiaq in bits and pieces in my school, while being scolded by my Aahna for not speaking it well enough and being taunted on the playground for speaking it at all. When I reached the University of Wisconsin—Madison as an undergraduate, I tried to assimilate into Midwestern culture as quickly as possible, while being called on to represent all 500 tribes by professors who did not distinguish between Prairie tribes and Inupiaq.

I stumbled into research as an undergrad through a work-study program in a plant pathology lab in an attempt to pad my CV for medical school. Medical school was most promising at the time because I had no idea that people still performed research. This second job (my “real” job was as a nursing assistant, which paid much better) opened my eyes to a world in which glassware was immaculate, the smell of broth penetrated my clothes, and no one asked me to speak on behalf of all natives.

I switched labs a couple of times during undergrad, getting eaten alive by mosquitoes one summer in a vector biology lab before settling into a lab to study toxin production in Staphylococcus aureus. Given that I was still very interested in clinical microbiology, this research seemed Important To Many People.

During graduate school, I explored parasitology, influenced heavily by an undergraduate parasitology lecture and lab from Bruce Christensen and Janet Schrader. The complex life cycles and multiple effects on various hosts that eukaryotic organisms have seemed more exciting than the comparatively simple life cycles of bacteria. My principal investigator, Laura Knoll, was honest and up front about the complications of being a woman in STEM, balancing life and work, and gave important glimpses into the life of a tenured faculty member. Still, when she asked what I wanted to do with my life, I told her frankly “I want your job.”

At the end of graduate school, I heard a series of talks on host-microbe interactions and was intrigued by the idea that microbes could benefit the host, not just damage it. I read a series of papers by Arturo Casadevall and Liise-anne Pirofski redefining the terms we use for host-microbe interactions. This spurred me to ask Margaret McFall-Ngai, whom I deeply respect in this field, for a list of suggestions of faculty members to do a postdoc with. She recommended Karen Guillemin of the University of Oregon, who uses both zebrafish and fruit flies to study host-microbe interactions.

Dr. Guillemin is not just a researcher in host-microbe interactions, however; she was the leader of a group using systems biology to explore several aspects of host-microbe interactions. She brought together mathematicians, physicists, ecologists, evolutionary biologists, neuroscientists, and others to build new microscopes, develop new protocols for deriving gnotobiotic organisms, and develop other tools to examine the system as a whole. This was revolutionary to me, as I had always focused on single pathways or genes or environmental stimulants. I will forever be indebted to her for the rich education she gave me not just in host-microbe interactions but also in systems biology and interdisciplinary science, as well as to my co-mentor Bill Cresko, for teaching me the value of using an evolutionary model organism for host-microbe interaction studies.

Being in the Lower 48 for 20 years was difficult as an Inupiaq. For most of my time at UW–Madison, I was the only Inupiaq on campus. Of 40,000 students, only 250 were American Indian/Alaskan Native, and I met few of them until I became more involved in the Native American student groups Wunk Sheek and AISES as a graduate student. I had no one to speak Inupiaq with, so I completely lost the few words I knew. My folks sent occasional care packages, but the seal oil containers broke in transit and I didn’t have anyone to share muktuk with who would understand the hard work and value of the precious food. I worried that the smell of dried caribou would offend my labmates, so I ate it only occasionally. Importantly, I was not studying questions that were important to Inupiaq. Few of the people from home suffered from Toxoplasma gondii or Staphylococcus aureus infections, so trying to explain the value of the research I was doing was difficult.

My aunt Louvie Harris, who passed away when I was in graduate school, was a major influence in my drive to look for questions important to Inupiaq. She supported me when I was trying to learn Inupiaq from a dictionary, she told me the Inupiaq traditions to use when giving birth, and she reminded me of the plants that we used for thousands of years to cure everything from colds to cancer.

When I became an assistant professor at the University of Alaska Anchorage, I stepped back into a world filled with Alaskan Natives. Now, rather than asking “what are you studying,” the questions became “how does your work help Alaskan Natives?” Although I had been asking that question of myself since undergraduate school, now I can ask Alaskan Natives, including Inupiaq elders, about the questions that are important to Alaskan Natives. I focus on how to use traditional plants, like the tundra tea I collected with my dad this summer, to treat microbial infections that affect our people and how to ameliorate the effects that contaminants have on the ecosystem using bioremediation. Now I am closer to being one: Dr. Kat, Inupiaq and researcher.

